# A pathogenesis-related protein 1 of Cucurbita moschata responds to powdery mildew infection

**DOI:** 10.3389/fgene.2023.1168138

**Published:** 2023-08-01

**Authors:** Wei-Li Guo, He-Lian Yang, Jin-Peng Zhao, Shi-Jie Bian, Yan-Yan Guo, Xue-Jin Chen, Xin-Zheng Li

**Affiliations:** ^1^ School of Horticulture and Landscape Architecture, Henan Institute of Science and Technology, Xinxiang, China; ^2^ Henan Province Engineering Research Center of Horticultural Plant Resource Utilization and Germplasm Enhancement, Xinxiang, China

**Keywords:** PR-1, *Cucurbita moschata*, powdery mildew, transgenic tobacco, functional analysis

## Abstract

Pumpkin (*Cucurbita moschata* Duch.) productivity is severely hindered by powdery mildew (PM) worldwide. The causative agent of pumpkin PM is *Podosphaera xanthii*, a biotrophic fungus. Pathogenesis-related protein 1 (PR1) homolog was previously identified from transcriptomic analysis of a PM-resistant pumpkin. Here, we investigated the effects of *CmPR1* gene from pumpkin for resistance to PM. Subcellular localization assay revealed that *CmPR1* is a cytoplasmic protein in plants. The expression of *CmPR1* gene was strongly induced by *P. xanthii* inoculation at 48 h and exogenous ethylene (ET), jasmonic acid (JA) and NaCl treatments, but repressed by H_2_O_2_ and salicylic acid (SA) treatments. Visual disease symptoms, histological observations of fungal growth and host cell death, and accumulation of H_2_O_2_ in transgenic tobacco plants indicated that *CmPR1* overexpression significantly enhanced the resistance to *Golovinomyces cichoracearum* compared to wild type plants during PM pathogens infection, possibly due to inducing cell death and H_2_O_2_ accumulation near infected sites. The expression of *PR1a* was significantly induced in transgenic tobacco plants in response to *G. cichoracearum*, suggesting that *CmPR1* overexpression positively modulates the resistance to PM via the SA signaling pathway. These findings indicate that *CmPR1* is a defense response gene in *C. moschata* and can be exploited to develop disease-resistant crop varieties.

## 1 Introduction

Cucurbita powdery mildew (PM) is one of the most destructive diseases that acutely diminishes the productivity and quality of pumpkin crops globally. The causal agent of PM in pumpkin is *Podosphaera xanthii*, a biotrophic fungus widely distributed worldwide (Perez-Garcia et al., 2009; Fukino et al., 2013). The use of pesticides control PM is associated with premature leaf senescence in pumpkin and the risk of developing drug-resistant pathogens due to prolonged usage. Also, pesticides cause environmental pollution, which poses a health risk to animals and humans. Most cultivated pumpkin varieties are very susceptible to PM, especially when grown at high temperatures under dry and wet alternating conditions (Mccreight, 2003). Therefore, there is a need to screen for PM-resistant genes to accelerate the development of new pumpkin varieties with PM resistance.

PR1 (pathogenesis-related protein 1) proteins play a crucial role in plant defense responses. For example, PR1 accounted for about 2% of the total protein in tobacco leaves infected by pathogens (Alexander et al., 1993). PR1 proteins are grouped under a multigene superfamily known as CAP (cysteine-rich secretory protein, antigen 5, and pathogenesis-related-1 protein) (Gibbs et al., 2008) and can be broadly categorized as acidic or alkaline based on their theoretical isoelectric points. Most PR1 proteins contain stress signaling peptides such as CAPE-1 (CAP-derived peptide 1), which comprise the last 11 amino acids from the C-terminus of the PR1 protein (Chen et al., 2014). Plant PR1 is typically considered an indicator of salicylic acid (SA) inducible systemic acquired resistance (SAR), a plant immune response that prevents further spread of infection to non-infected parts of the host plant. SAR also plays a crucial role in hypersensitive response (HR)-related cell death (Van Loon et al., 2006). Recent reports have emerged demonstrating that PR1 genes in various plants are involved in the response to many phytopathogens attack (Soliman et al., 2019; Han et al., 2023), especially fungi (Anisimova et al., 2021; Kiba et al., 2007). PR1 proteins are involved in sterol-binding activity of caveolin-binding motif (CBM) in the CAP region, which targets and inhibits phytopathogens in response to host infection (Breen et al., 2017; Gamir et al., 2017; Wang et al., 2022). PR1 proteins also facilitate cell wall thickening to limit the invasion and spread of pathogens in the apoplast (Wang et al., 2013). PR proteins have been extensively applied in the development of transgenic plants with broad-spectrum resistance to a wide range of pathogens. However, there are only a few studies on the function of PR1 proteins in *C. moschata* or related Cucurbita species during biotic stress responses.

In an earlier study, we identified pumpkin genotypes with high powdery mildew resistance through natural and artificial inoculations under field conditions (Zhou et al., 2010). Moreover, transcriptome sequencing revealed variations in the expression levels of many PR genes in pumpkin in response to *P. xanthii* infection (Guo et al., 2018). To clarify the role of PR proteins in response to *P. xanthii* infection, we isolated a PR1-like gene from pumpkin and named it *CmPR1* (GenBank accession no. MH105818). Additionally, the *CmPR1* expression profile and the subcellular localization of the encoded protein were analyzed. Finally, we overexpressed the *CmPR1* gene in tobacco to validate its function in disease resistance.

## 2 Materials and methods

### 2.1 Plant material and stress treatments

Seeds of two pumpkin genotypes inbred line “112–2” and cultivar “JJJD” were provided by Henan Institute of Science and Technology, Henan Province, China. The inbred line “112–2” is highly resistant to *P. xanthii*, whereas cultivar “JJJD” is susceptible. The pumpkin seeds were sown in plastic pots (9 cm deep) containing a 3:1 mixture of grass charcoal and perlite and the resulting seedlings were grown as previously described by Guo et al. (2018). The seedlings at the fourth leaf stage (at approximately 4 weeks) were treated as previously described (Guo et al., 2019). In detail, the seedlings were sprayed with freshly-prepared spore suspension (10^6^ spores/mL) and solutions containing 1.5 mM H_2_O_2_, 100 mM SA, 100 mM abscisic acid (ABA), 100 mM methyl jasmonate (MeJA), 0.5 g/L ethephon (Eth), or 0.4 mM sodium chloride (NaCl) in separate treatments. Distilled water alone was used as the control treatment. Specially, SA was dissolved in 10% ethanol, and MeJA, Eth in sterile water. Control plants were sprayed with 10% ethanol and sterile water, individually (Tang et al., 2017). The treated seedlings were maintained in a growth chamber with a photoperiod of 15 h/9 h light/dark (28°C/18°C, 5,500 lux light intensity). Subsequently, two or three upper leaves of four separate seedlings were sampled to detect *CmPR1* expression profiles at 0, 3, 6, 12, 24, and 48 h time points under various stress conditions. The experiments were arranged in a completely random block design with three biological replicates.

### 2.2 *CmPR1* cloning and sequence analysis

The PR1 EST (GenBank accession No. SRR5369792) was isolated from a transcriptome of pumpkin seedlings inoculated with *P. xanthii* pathogens (Guo et al., 2018). Full-length open reading frame (ORF) of PR1 was cloned using cDNA sequence as a probe by a homology-based candidate gene method. ExPASy ComputepI/Mw tool was used to calculate the theoretical molecular weight (Mw) and isoelectric point (pI) of *CmPR1* protein. DNAMAN tool (version 6.0.40) was used to analyze sequence alignment of amino acids between pumpkin *CmPR1* and other homologous PR1 proteins. Blast tool of the NCBI databases was used to search for homologous protein sequences (http://www.ncbi.nlm.nih.gov/blast). SignalP5.0 server was utilized to predict potential signal peptide regions and the cleavage sites.

### 2.3 Subcellular localization analysis

The *CmPR1* ORF was inserted into the pBI221-GFP vector to form a green fluorescent protein (GFP)-labeled *CmPR1* fusion construst. Transient expression of the *CmPR1*-GFP fusion gene in *Arabidopsis thaliana* protoplasts was analyzed using polyethylene glycol method described by Lee et al. (2013). GFP fluorescence was directly imaged using a confocal fluorescence microscope (UltraVIEWVoX, Olympus, Japan) under the excitation wavelength of 488 nm and the capture wavelength of 448–508 nm. Cell membrane and nucleus were detected by 1,10-dioctadecyl-3,3,30,30 -tetramethylindocarbocyanineperchlorate (Dil) and 2-(4-Amidinophenyl)-6-indolecarbamidine dihydrochloride (DAPI) staining, respectively.

### 2.4 Plasmid vector construction and genetic transformation of tobacco

Full-length forward and reverse primers of *CmPR1* gene containing *Bam*HI and *Kpn* I sites, respectively, were used to amplify the cDNA fragment. The cDNA fragment was inserted into the cloning site of the pMD19-T vector (Takara, Japan) and then digested from the recombinant pMD19-T vector using restriction endonuclease *Bam*HI and *Kpn* I. The *CmPR1* cDNA fragment with the restriction enzyme sites was ligated to the *Bam*HI-*Kpn* I site of the expression vector pVBG2307 harboring 35 S cauliflower mosaic virus promoter. The recombinant vector pVBG2307-*CmPR1* was validated by sequencing and double enzyme digestion and then transformed into *Agrobacterium tumefaciens* GV3101 by electroporation. Putative transgenic tobacco (*Nicotiana tabacum* L. cv. NC89) plants overexpressing *CmPR1* were generated via Agrobacterium-mediated transformation using the leaf disk method (Li et al., 2012). Subsequently, transgenic plants were identified by the kanamycin selective medium followed by PCR amplification of reporter gene *NPTII* and target gene *CmPR1*. More than three homozygous T2 transgenic lines were used for subsequent experiments.

### 2.5 Evaluation of disease resistance of transgenic tobacco overexpressing *CmPR1*

Transgenic tobacco plants overexpressing *CmPR1* were used to analyze resistance to powdery mildew at the six-leaf stage. The causative agent of tobacco powdery mildew (*Erysiphe cichoracearum* DC) was collected from naturally infected tobacco leaves. Spore suspension (10^6^ spores/mL) of the pathogen was sprayed onto asymptomatic plants. Subsequently, various parameters were assessed, including disease incidence, growth of the PM fungus, H_2_O_2_ accumulation and cell death. The inoculated leaves *in vitro* were examined for the disease symptoms of powdery mildew (Guo et al., 2019). After PM inoculation, the petioles were wrapped with absorbent cotton wool and put into plastic box with a lid to moisturize. The inoculated plants were cultured at 28°C in light/dark (16 h/8 h). Attention was paid to note the incidences of moisture addition during the period.

The infected leaves of transgenic plants and wild type (WT) were symmetrically excised along the sides of the main vein at different corresponding time points after pathogen inoculation. Subsequently, histological observations of fungal growth and host responses were performed as described by Guo et al. (2018). The growth of PM fungus and cell death around the infection sites were examined by methyl blue staining and trypan blue staining, respectively. H_2_O_2_ accumulation was detected by 3, 3-Diaminobenzidine (DAB) staining (Choi et al., 2012). At least 20 infection sites were examined in each of four randomly selected leaf parts in each experiment.

### 2.6 Gene expression analysis

Total RNA was extracted from pumpkin and tobacco leaves using the Trizol method (Invitrogen, United States) according to the manufacturer’s instructions. Total RNA concentration and purity (OD260/OD280 and OD260/OD230 ratio) were measured to detect the quality. RNA degradation was examined using 1% agarose gel electrophoresis. The first strand of cDNA was synthesized from total RNA as a template using PrimeScript™ II 1st Strand cDNA Synthesis Kit (TaKaRa, Japan). Subsequently, the cDNA was used as a template for qRT-PCR, which was performed on aniCycleriQTM Multicolor PCR Detection System (Bio-Rad, United States) using TB Green™ Premix Ex Taq™ II (Tli RNaseH Plus) (Invitrogen, United States). Relative gene expression levels were calculated using the 2^−ΔΔCT^ method. *CmPR1* gene expression was analyzed to test its response to pathogen infection and exogenous signal molecules. In addition, the expression of marker genes (*NtNPR1*, *NtPR1a*, *NtPR5*, *NtPDF1.2*, and *NtPAL*) associated with SA or JA/ET signal transduction was examined to evaluate the defense response mechanism of tobacco under powdery mildew infection. Pumpkin *β -actin* gene (Wu and Cao, 2010) and tobacco *NtEF1-α* gene (Xiang et al., 2017) were used as internal controls for normalization of gene expression. All primers used in this study are listed in Supplementary Table S1.

### 2.7 Statistical analysis

Data values are expressed as mean ± standard error (SE). Differences between various treatments were analyzed using one-way analysis of variance (ANOVA) and means separated by LSD test (*post hoc*) at a *p*-value ≤0.05. Data analysis was performed using IBM SPSS Statistics 25 software (SPSSInc., United States).

## 3 Results

### 3.1 Isolation and analysis of *CmPR1* gene from pumpkin

*CmPR1* was cloned based on the transcriptome of a highly resistant pumpkin genotype inoculated with powdery mildew using PR1 EST sequence as a probe. The full length of the *CmPR1* gene (GenBank:MH105818) was 658 bp, translating to 198 amino acids with a molecular weight of 22.9 KD and a pI of 6.88. Based on NCBI conserved domains database and SMART database, *CmPR1* protein contained a CAP superfamily domain structure, a signal peptide, and SCP domains. Sequence alignment of amino acids between pumpkin *CmPR1* and other plant genes showed that *CmPR1* amino acids were highly identical to those of *C. maxima* (98.9% identical), *C. pepo* (96.5% identical), *Benincasa hispida* PR1 (91.2% identical), and *Cucumis sativus* PR1 (90.7% identical). In addition, those proteins contained highly conserved CAP domain sequences (amino acids 58–194), confirmed to belong to the CAP superfamily. A SignalP analysis revealed signal peptide regions (amino acids 1–21) of PR1 proteins at the N terminal in alignment. The predicted cleavage site is indicated by a circle (Supplementary Figure S1).

**FIGURE 1:**
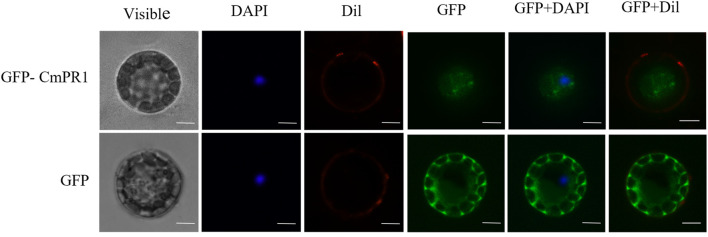
Subcellular localization of *CmPR1* protein in Arabidopsis protoplast. The fused GFP-*CmPR1* constructs were introduced and transiently expressed in Arabidopsis protoplast. Scale bars = 5 µm. Note that images of GFP alone were duplicated with those described in our previous article because we had simultaneously expremented subcellular localization of *CmPR1* together with CmSGT1 proteins (Guo et al., 2019).

The deduced *CmPR1* protein contains a predicted signal peptide and is potentially a secreted protein. The GFP signal in Arabidopsis protoplasts expressing GFP alone was distributed in the cytoplasm and nucleus. However, the *CmPR1*-GFP fusion protein was transiently expressed through out the cell (Figure 1). These results suggest that *CmPR1* is a cytoplasmic protein.

### 3.2 *CmPR1* expression analysis

**FIGURE 2:**
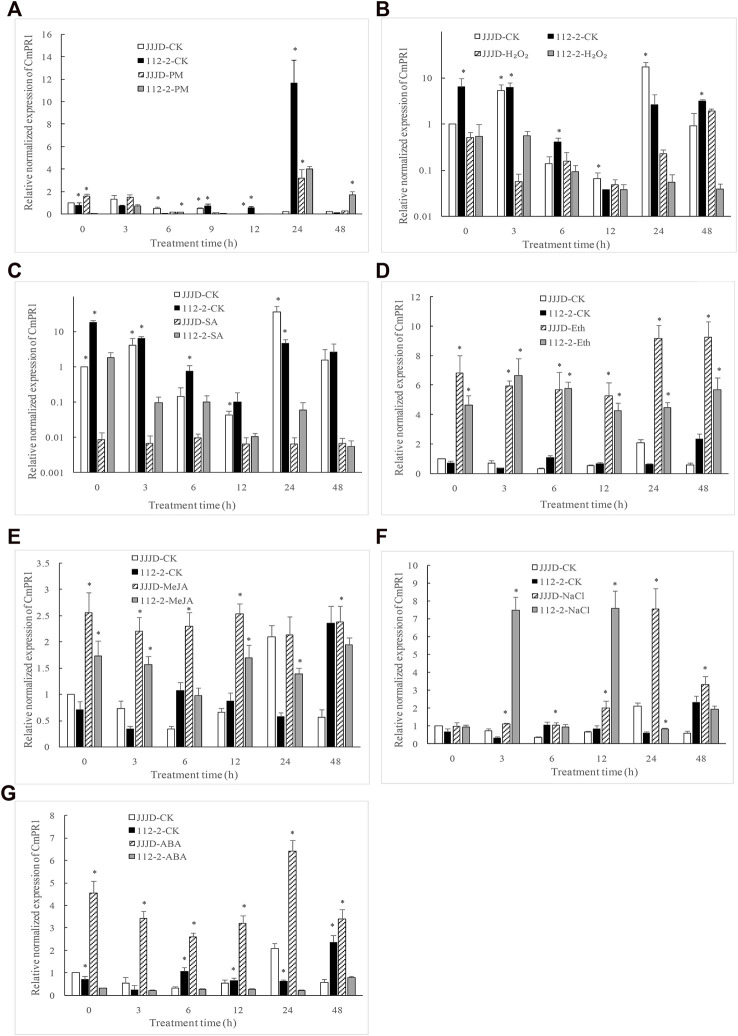
Expression level of *CmPR1* gene in leaves of pumpkin plant after exogenous treatments. The seedlings were sprayed with a spore suspension of *Podosphaera xanthii* **(A)**, exogenous hydrogen peroxide (H_2_O_2_) **(B)**, salicylic acid (SA) **(C)**, ethephon (Eth) **(D)**, methyl jasmonate (MeJA) **(E)**, NaCl **(F)**, and abscisic acid (ABA) **(G)**. The asterisks denote statistical significance between treatments (JJJD-CK vs. JJJD-treatment and 112-2-CK vs. 112-2-treatment) at *p* < 0.05.

Compared with control, *CmPR1* expression was significantly downregulated (0.13 fold) at 9 h, 12 h, and 24 h, but significantly upregulated (19.2 fold) at 48 h in resistant 112–2 seedlings inoculated with PM pathogens (Figure 2), indicating that *CmPR1* was responsive to *P. xanthii* infection. Notably, H_2_O_2_ (Figure 2B) and SA (Figure 2C) treatments significantly inhibited *CmPR1* expression in control and inoculated seedlings. Specifically, *CmPR1* expression decreased by 0.02 fold in control under H_2_O_2_ treatment for 24 h and by about 0.05 fold in control under SA treatment, suggesting that *CmPR1* potentially plays a negative regulatory role in response to exogenous SA and H_2_O_2_ treatments. An opposite *CmPR1* expression pattern was observed in the control and inoculated seedlings following Eth (Figure 2D), MeJA (Figure 2E), and salt stress (Figure 2F) treatments. In particular, *CmPR1* expression increased by 16 fold in control at some time points after Eth treatment, indicating that *CmPR1* potentially responds to salt stress via ethylene (ET) and MeJA signaling pathways. *CmPR1* expression was lower in resistant “112–2” seedlings under ABA treatment (Figure 2G), but was significantly higher in susceptible “JJJD” seedlings than in control, suggesting that the effect of ABA on *CmPR1* gene expression may be related to pumpkin materials.

### 3.3 Response of transgenic tobacco overexpressing *CmPR1* to PM infection

**FIGURE 3:**
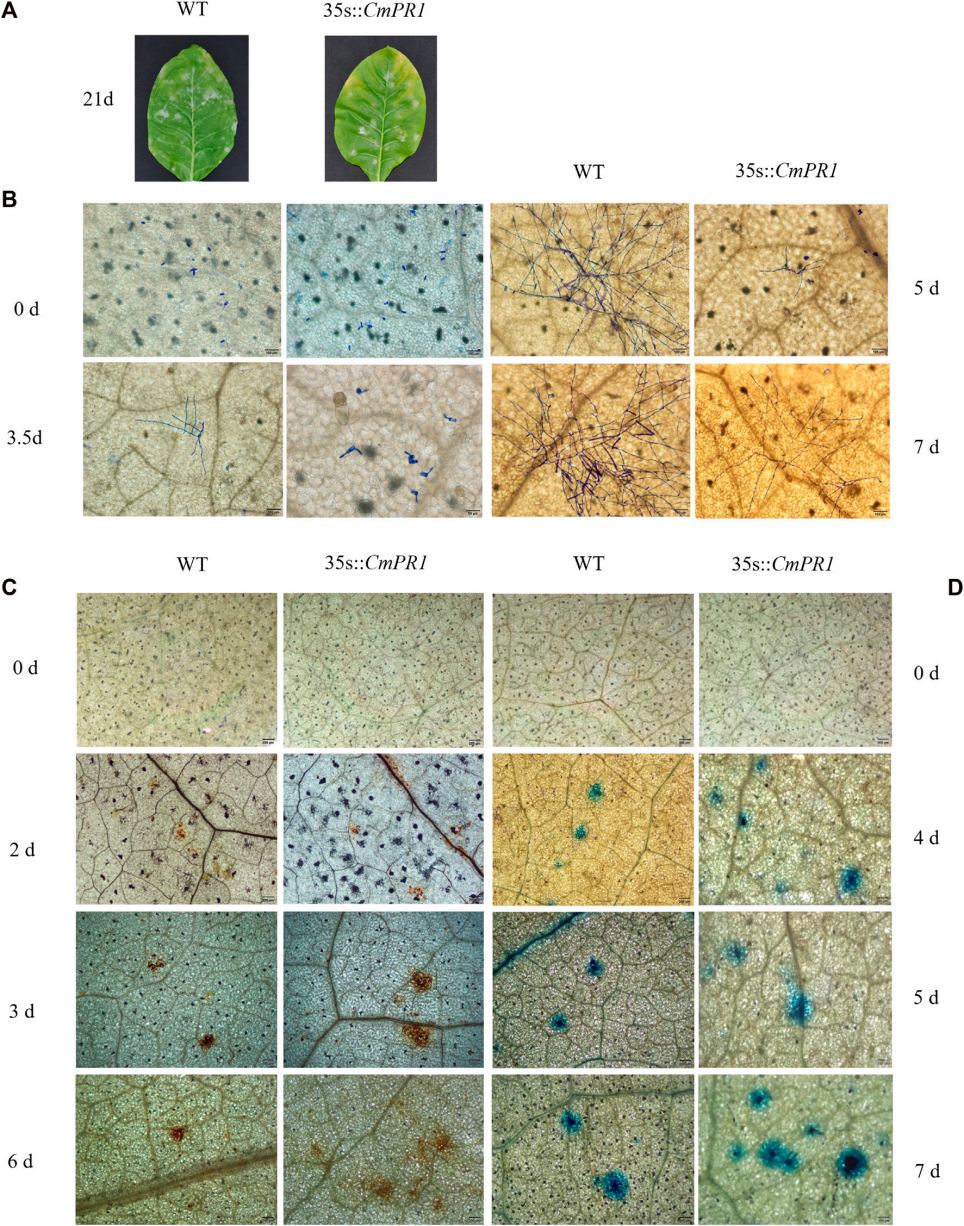
Resistance analysis of tobacco leaves infected with *Erysiphe cichoracearum*. The pathogenic symptoms of transgenic tobacco (35 s::*CmPR1*) and wild type (WT) after 21 d of infection, scale bars = 2.0 cm **(A)**; spores growth of the infected leaves **(B)**; DAB-stained infected-leaves **(C)**; Trypan blue-stained infected-leaves **(D)**. Scale bars = 200 mm.

*CmPR1* expression was upregulated in transgenic tobacco plants but undetectable in the WT plants under normal conditions (Supplementary Figure S2), indicating that *CmPR1* was overexpressed in the transgenic plants. Regarding disease symptom manifestation, white powdery areas in the transgenic plants were slightly less severe than in the WT plants after 21 d post-inoculation with PM (Figure 3A). Based on microscopic analysis of the fungal growth, no geminated *P. xanthii* conidia were detected on both transgenic and WT plants at 0 dpi, conidia began to germinate and differentiate into germtubes at 3.5 dpi, powdery mildew mycelia appeared at 5 dpi, and bifurcated formed secondary mycelia at 7 dpi in the inoculated transgenic leaves (Figure 3B). However, in the inoculated WT leaves, conidia germinated and grew into mycelia at 3.5 dpi, dense hyphal network developed at 5 dpi, and chains of conidia formed at 7 dpi. These observations indicate that the growth and proliferation of the powdery mildew fungus were weaker in the transgenic plants than in the WT plants.

The DAB-stained brown spots in inoculated transgenic plants appeared at 2 dpi, expanded and deepened in color at 3 dpi, and continued to increase in continuous patches at 6 dpi (Figure 3C). The DAB-stained brown spots were more in number and area in transgenic plants than in WT plants, and the differences were more obvious with the prolongation of infection time. In addition, the sporadic stained blue spots in the transgenic plants abounded at 4 dpi and continued to expand and increase between 5 and 7 dpi, becoming more and larger than in the WT plants (Figure 3D). Overall, these results suggest that *CmPR1* overexpression in tobacco plants enhances reactive oxygen species accumulation and HR-cell death in response to powdery mildew.

### 3.4 Expression analysis of hormone signaling-related genes in tobacco

**FIGURE 4:**
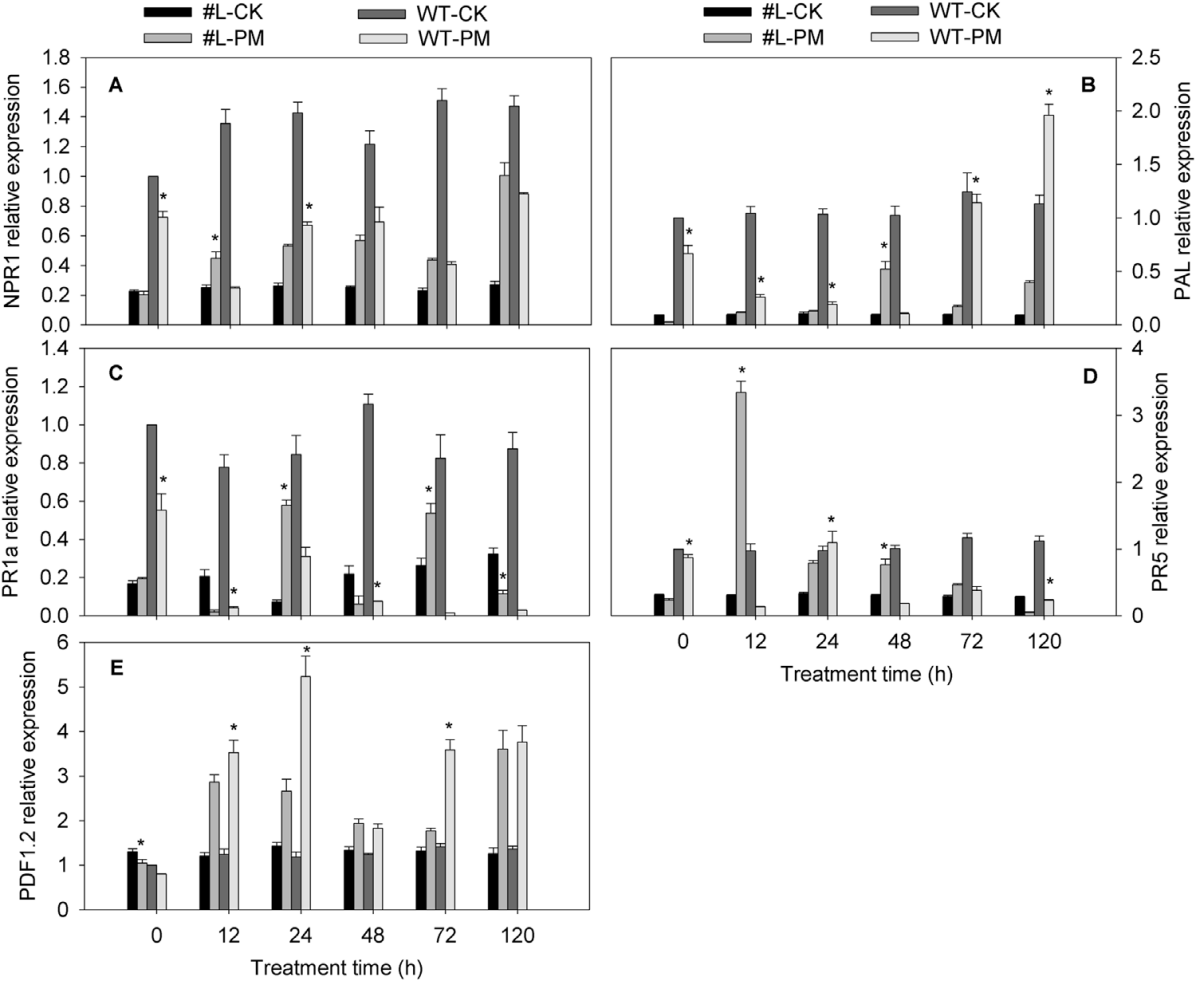
Expression analysis of hormones signaling-related genes in tobacco infected with *Erysiphe cichoracearum*. The equally-mixed samples of three transgenic tobacco lines (#L) and wild type (WT) plants were used to analyze genes expression by qRT-PCR. **(A)**, *NPR1*; **(B)**, *PAL*; **(C)**, *PR1a*; **(D)**, *PR5*; **(E)**, *PDF1.2*. Asterisks denote significant difference between transgenic lines and WT plants (#L-PM vs. WT-PM and #L-CK vs. WT-CK) at *p* < 0.05.

The *NPR1*, *PAL* (except at 0 h), and *PR5* (except at 120 h) expression level was higher in the PM-infected transgenic plants than in the CK plants, whereas these *NPR1*, and *PR5* (except at 24 h) genes level was lower in the PM-infected WT plants than in the CK plants. The *PDF1.2* expression levels were higher in the transgenic and WT plants infected with PM than in the CK plants, implying that PM induced the expression of this gene. The *PR1a* expression levels in the PM-infected WT plants were lower than in the CK plants, and the irregular pattern of this gene expression was observed in the transgenic plants infected with PM compared with CK plants. Under PM inoculation, the expression of *PDF1.2* and *PAL* (except 48 h) were wholly lower in the transgenic lines than in WT plants (Figure 4). However, compared with WT-PM treatment, expression of *PR1a* in the PM inoculated transgenic lines was signifcantly upregulated at 24 h, 72 h, and 120 h. Meanwhile, the expression of *PR5* in PM inoculated transgenic lines was significantly higher at 12 h and 48 h (24.7 and 4.1 fold of WT-PM), but slightly lower at 24 h and 120 h compared with WT-PM treatment. Notably, no significant difference in *NPR1* expression was observed between PM inoculated WT and transgenic lines. Altogether, these findings show that *CmPR1* overexpression in tobacco plants inhibits *PDF1.2* and *PAL* expression and induces *PR1a* expression in response to PM infection. Furthermore, the increased PM resistance of the transgenic plants appeared to be related to the upregulated expression of these genes.

## 4 Discussion

In this study, a novel pumpkin PR1-like gene (*CmPR1*) was cloned. The predicted amino acid contained a conserved CAP domain and a signal peptide, indicating that *CmPR1* is a putative pumpkin PR1 protein. *CmPR1* protein is presumed to be acidic based on pI data. As a fragment of protein, a signal peptide is typically located at the N terminal of secreted protein. Signal peptides determine the secretory characteristics of a protein and its localization in the cell. The results of this study show that *CmPR1* protein located in the cytoplasm, suggesting that it may be secreted into the cytoplasm to perform its biological functions through the N-terminal signal peptide. This is consistent with a previous study reported that PnPR-like protein is localized in the cytoplasm (Li et al., 2021).

Phytohormones, such as SA, JA, ET, and ABA are involved in regulating plant disease resistance. Expression of *BjPR1* gene from *Brassica juncea* was strongly induced by *Alternaria brassicae* infection and SA treatment, but not by JA or ABA treatments (Ali et al., 2018). *PnPR-1* transcripts from *Piper nigrum* were significantly upregulated during *Phytophthora capsici* infection (Kattupalli et al., 2021). In this study, *CmPR1* expression was significantly upregulated by late *Podosphaera xanthii* infection and exogenous application of ET and MeJA, but significantly inhibited by SA and H_2_O_2_ treatments. This contradicts some previous studies that showed acidic PR proteins are upregulated by various endogenous signaling molecules (ROS) and phytohormones (SA) in response to pathogen attack, while basic PR proteins are upregulated by ET and MeJA (Rigoyen et al., 2020). Our findings are consistent with some studies. For example, acidic *OgPR1a* expression level was strongly induced by exogenous ET and JA (Shin et al., 2014). Although most of the PR1 proteins reported by previous studies were alkaline, it is not recommended to simply judge whether PR1 could enhance disease resistance based on pI value and sequence homology without conducting disease bioassays (Li et al., 2011).

The RNase activity of soybean *GmPRP* (PR gene) restricts the mycelial growth of *Phytophthora sojae* (Xu et al., 2014). Also, the RNase activity of jelly fig *PR-4* is related to its inhibitory effect on pathogens growth (Lu et al., 2012). In the present study, conidium germination and mycelium growth were significantly restricted in the transgenic tobacco plants expressing *CmPR1*, reflecting the role of *CmPR1* in enhancing disease resistance. However, further studies should be conducted to determine whether the inhibitory effect of *CmPR1* protein on powdery mildew growth is related to RNase activity.

Plants initiate various defense responses during a pathogen attack. The first line of defense typically involves structural responses that include cell wall strengthening, and waxy epidermal cuticle development, and production of antimicrobial molecules (Hakim et al., 2018; Manghwar and Hussain, 2022). However, many pathogens have evolved mechanisms to break the plant’s first defense barrier; when this occurs, plants activate an alternative defense pathway, which is the metabolic modifications involving hypersensitive response, oxidative burst, synthesis of SA, ET, and JA, and eventually the synthesis of PR proteins (Das et al., 2011; Van Schie and Takken, 2014; Li et al., 2015). These defense responses are triggered based on the life style of the pathogen. For example, ROS generation and HR inhibit the growth of (hemi)biotrophs. On the contrary, necrotrophs stimulate ROS production to induce susceptibility-associated cell death of the host (Mengiste, 2012). In this study, DAB-staining revealed higher levels of H_2_O_2_ accumulation in the transgenic plants than in the WT plants. Excessive ROS accumulation induces cell death in plants (Petrov and Van Breusegem, 2012). Compared with inoculated WT plants, trypan blue staining showed that the transgenic plants exhibited more serious necrotic regions following the pathogen attack. This restricted the spread of the pathogen beyond the infection site, preventing further growth and spread to other plant parts. Overexpression of *CmPR1* in transgenic tobacco plants potentially limited the proliferation of powdery mildew pathogen by activating HR-related cell necrosis accompanied by ROS generation. In a similar study, TaTLP1 was shown to interact with TaPR1 to increase antifungal activity and inhibit fungal growth and cell death and H_2_O_2_ accumulation in TaTLP1-TaPR1-cosilenced plants were observed (Wang et al., 2020). Also, overexpression of *VpPR4-1* increased powdery mildew resistance of grape by repressing the growth of powdery mildew (Dai et al., 2016).

Plant PR proteins facilitate defense responses to microbial pathogens via direct or indirect pathways. A direct response against invading microbial pathogen is typically characterized by inhibition of pathogen growth or spore germination. On the other hand, an indirect response involves PR isoforms, which play a more crucial role in plant resistance against pathogens. Plant pathogen invasion is quickly followed by activation of defense signaling pathways such as SA and JA, which induces the accumulation of PR proteins to minimize pathogen load or disease onset in uninfected plant organs. At intermediate SA levels, NPR1 (nonexpresser of PR1) accumulates and interacts with the TGA transcription factor, functioning as a coactivator of SA-responsive genes, including PR genes (Caarls et al., 2015). The *PAL*, *PR1a*, and *PR5* expression levels are markers of the SA signaling pathway. The *PDF1.2* gene is important for the JA/ET-dependent signaling pathway. In this study, following a PM infection, the *PR1a* expression level was higher in the transgenic plants than in the WT plants, whereas the opposite pattern was observed for the *PAL* and *PDF1.2* expression levels. This suggests that in the SA pathway, the transactivation of *PR1a* is dependent on *CmPR1*, whereas the transactivation of *PAL* is unaffected by *CmPR1*. Additionally, *CmPR1* does not directly affect the JA/ET-dependent defense pathway to regulate *PDF1.2* expression. Notably, *CmPR1* overexpression in tobacco alleviated the symptoms of tobacco powdery mildew by activating SA defense signaling pathways without inducing *PAL* expression level and suppressing the activities of JA/ET-dependent defense pathways. This is consistent with previous studies that have revealed that the resistance to biotrophic and hemibiotrophic pathogens is often driven by SA signaling, while resistance to necrotrophic pathogens is mediated by JA and ET signaling (Pieterse et al., 2012; Guo et al., 2019).

## 5 Conclusion

The results of this study indicate that heterologous expression of *CmPR1* gene from pumpkin enhances the resistance of transgenic tobacco plants to PM. Additionally, we revealed that overexpression of *CmPR1* gene in tobacco plants decreases the development of mildew symptoms caused by E. cichoracearum, possibly by inducing ROS accumulation and HR near infected sites, which further activates SA defense signaling pathway in uninfected leaves. This study provides new insights into understanding the defense mechanisms of Cucurbita crops against fungal diseases and can be exploited to develop disease-resistant crop varieties.

## Data Availability

The datasets presented in this study can be found in online repositories. The names of the repository/repositories and accession number(s) can be found in the article/Supplementary Material.
