# Supplementary material for: A pathogenesis-related protein 1 of Cucurbita moschata responds to powdery mildew infection
Source: Front Genet. 2023 Aug 1;14:1168138. doi: 10.3389/fgene.2023.1168138 (PMC10427922; doi:10.3389/fgene.2023.1168138)
Supplement: Supplementary file 1 [file Table1.pdf]

Supplementary Table 1 Primers designed in this study

Gene	Accession	Primer sequence(5’-3’)
CmPR1	MH105818	F: TAGGCAGAAGCAAGCAACAATAA<br>R: ACATAGGCGAGCGGCCCTACTAA
RT-qPCR for *CmPR1*		F: TAGGCAGAAGCAAGCAACAATAA<br>R: CCCTGTAGATGGTTTCGTTGTCC
NtNPR1	U76707	F: ACATCAGCGGAAGCAGTAG<br>R: GTCGGCGAAGTAGTCAAAC
NtPR1a		F: CCTCGTACATTCTCATGGTCAAT<br>R: CCATTGTTACACTGAACCCTAGC
NtPR5		F: CCGAGGTAATTGTGAGACTGGAG<br>R: CCTGATTGGGTTGATTAAGTGCA
NtPDF1.2	T04323	F: GGAAATGGCAAACTCCATGCG<br>R: ATCCTTCGGTCAGACAAACG
NtPAL	X95342	F: GTTATGCTCTTAGAACGTCGCCC<br>R: CCGTGTAATGCCTTGTTTCTTGA
NtEF1-α	AF120093	F: TGTGATGTTTTTGTTCGGTCTTTAA<br>R: TCAAAAGAAAATGCAGACAGACTCA
β-actin		F: TCTCTATGCCAGTGGTCGTA<br>R: CCTCAGGACAACGGAATC
NPTII		F: AGACAATCGGCTGCTCTGAT<br>R: TCATTTCGAACCCCAGAGTC
