# Supplementary material for: A pathogenesis-related protein 1 of Cucurbita moschata responds to powdery mildew infection
Source: Front Genet. 2023 Aug 1;14:1168138. doi: 10.3389/fgene.2023.1168138 (PMC10427922; doi:10.3389/fgene.2023.1168138)
Supplement: Supplementary file 2 [file DataSheet1.pdf]

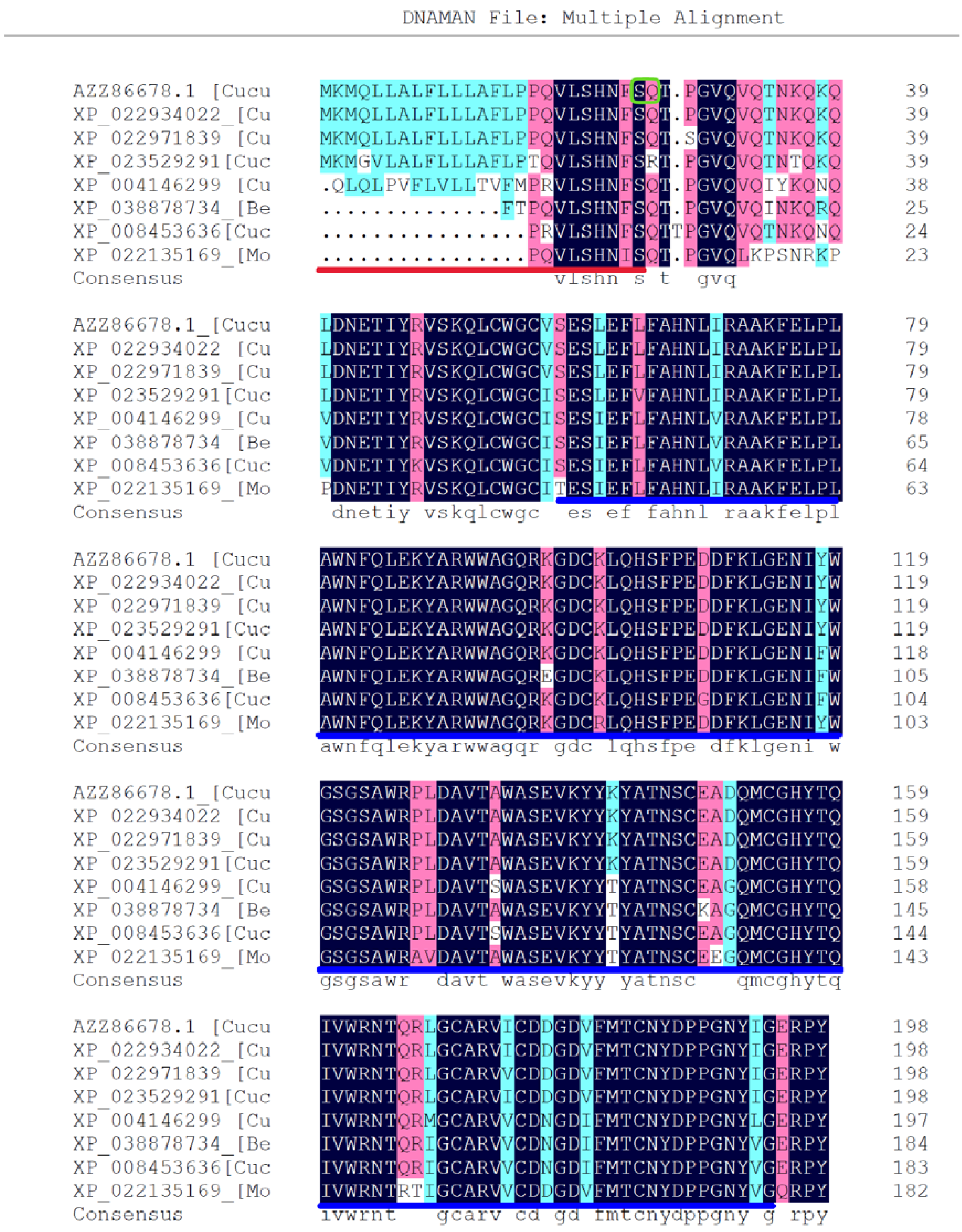

Supplementary Fig. 1 Multiple sequences alignment of pumpkin CmPR1 amino acid with its homologs of different plant species. Origin of sequences: AZZ86678.1 [*Cucurbita moschata* cv. '112-2'], XP_022934022 [*Cucurbita moschata* cv. 'Rifu'], XP_022971839 [*Cucurbita maxima*], XP_023529291[*Cucurbita pepo*], XP_004146299 [*Cucumis sativus*], XP_038878734 [*Benincasa hispida*], XP_008453636 [*Cucumis melo*], XP_022135169 [*Momordica charantia*]. The red colored underline indicates the signal peptide regions; the cleavage sites predicted are shown by the green circular. The blue underlines indicate the CAP domain structure of ~140 bp.

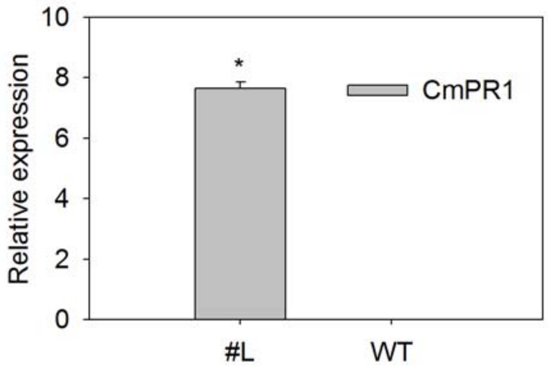

Supplementary Fig. 2 The expression of *CmPR1* in tobacco plants under normal growth conditions. #L represents the equally-mixed samples of three independent *CmPR1*-overexpressed transgenic lines. Asterisks denote statistical significance between both materials at $p < 0.05$.
